# Femoral malrotation after trochanteric fracture nailing: what is the safety zone of limb rotation during closed reduction?

**DOI:** 10.1007/s00402-025-06019-z

**Published:** 2025-08-04

**Authors:** Jakub Maléř, Michal Buk, Martin Michna, Milan Hrazdíra, Radek Bartoška, Jiří Skála-Rosenbaum

**Affiliations:** 1 Department of Orthopaedics and Traumatology, Third Faculty of Medicine, Charles University, University Hospital Kralovske Vinohrady, Šrobárova 1150/50, 100 34 Prague, Czech Republic; 2 Faculty of Medicine, Charles University, Plzeň, Czech Republic; 3 Department of Radiodiagnostics, Third Faculty of Medicine, Charles University, University Hospital Kralovske Vinohrady, Prague, Czech Republic

**Keywords:** Trochanteric fracture, Malrotation, Rotational error, Intramedullary nailing, Fracture reduction

## Abstract

**Introduction:**

Improper closed trochanteric fracture reduction can cause rotational malposition which can lead to serious consequences. The primary objective of this study was to assess the hypothetical threshold at which excessive rotation becomes hazardous due to a significant postoperative malrotation.

**Materials and methods:**

We conducted a prospective study focused on closed reduction in intramedullary nailing of trochanteric fractures (AO 31A1-3) in 100 consecutive patients and its influence on final malrotation. Immediately after the closed reduction, the rotation of affected limb was measured using a balanced goniometer and the values were compared to the postoperative CT calculation. Final femoral malrotation exceeding 15° was considered significant. All results were statistically analyzed.

**Results:**

In total we observed femoral malrotation exceeding 15° in 33 patients (33.3%). Internal rotation was significantly more common than external rotation (31 vs. 2 patients). Intraoperative rotation up to 15° resulted in a malrotation of 10.3% (3/29 patients). When limb rotation on the traction table exceeded 20°, malrotation incidence increased to 51.0% (26/51 patients), making this fixed position a risk factor for significant femoral malrotation (*p* = 0.0076). General anesthesia was also associated with a significantly higher rate of malrotation compared to spinal anesthesia (*p* = 0.0154), however we did not find any statistical difference in error rates based on patient BMI or physiological femoral neck ante-version.

**Conclusions:**

Our findings underscore the significant risk of femoral malrotation associated with perioperative rotations beyond 20°, emphasizing the need for precise rotational control during surgery. Excessive rotation on the traction table in an attempt to achieve better fracture alignment significantly increases the risk of femoral malrotation.

## Introduction

Trochanteric femoral fractures are among the most common fractures requiring surgery in the elderly population [1–3]. Complications associated with imperfect reduction are often discussed in the literature [4–7], but postoperative femoral malrotation is largely ignored, even when the risk of substantial malrotation exceeds 30% [8–12]. Over the few last decades, there has been increasing focus on femoral malrotation in shaft fractures resulting from suboptimal reduction [13–16]. A change in femoral rotation of more than 15° can lead to a variety of complications, including alterations in gait pattern contributing to limping, progression of osteoarthritis in surrounding joints, periarticular tendon pain, and overloading of the contralateral limb [13, 15, 17, 18]. Malrotations up to 15° are particularly well tolerated, especially in the elderly population.

Recently, a few studies have addressed femoral malrotation following hip fracture fixations. Some of these studies indicate that errors in femoral malrotation are far more common when compared to nailing of femoral shaft fractures. Failing to achieve proper reduction with subsequent femoral malrotation ranges from 20–40% of cases, making malrotation the most common error encountered in hip fracture fixations [8–12]. Many studies often deal with different risk factors attributed to different types of anesthesia, surgeon experience, or patient positioning during surgery, among others. However, none of these factors have been statistically confirmed so far. Recent studies also focus on surgical techniques aimed at minimizing the severity of malrotation [19–21]. Unfortunately, none of the aforementioned literature have provided us with a simple guideline applicable in routine practice for minimizing this error.

In our study, we focused on surgical technique on resulting malrotation. Specifically, we measured the rotation of the limb on the traction table during closed reduction in 100 consecutive patients. Based on comparison with postoperative CT we tried to identify a safe threshold of limb rotation at which significant deviation resulting in malrotation does not occur.

## Materials and methods

We conducted a prospective cohort study focused on CT analyses of malrotation following trochanteric fracture nailing (AO 31A1-3). This research encompassed 100 consecutive patients who underwent the procedure within a period of 12 months (I.-XII. 2023), constituting 25% of all trochanteric fractures fixations conducted in our department during that period. The average age of patients was 82 years (range 32–102), with the majority being women (72% vs. 28%).

Exclusion criteria consisted of patients with a history of any surgery on either one of the femurs, individuals with pathological fractures, those unable to participate in thorough postoperative examinations during their hospital stay, patients declining participation in the study and those subjected to open fracture reduction. After the procedure, participants underwent a specialized low-dose CT scan of the femurs. The study received approval from the hospital’s Ethics Committee and all patients provided informed consent.

**Fig. 1:**
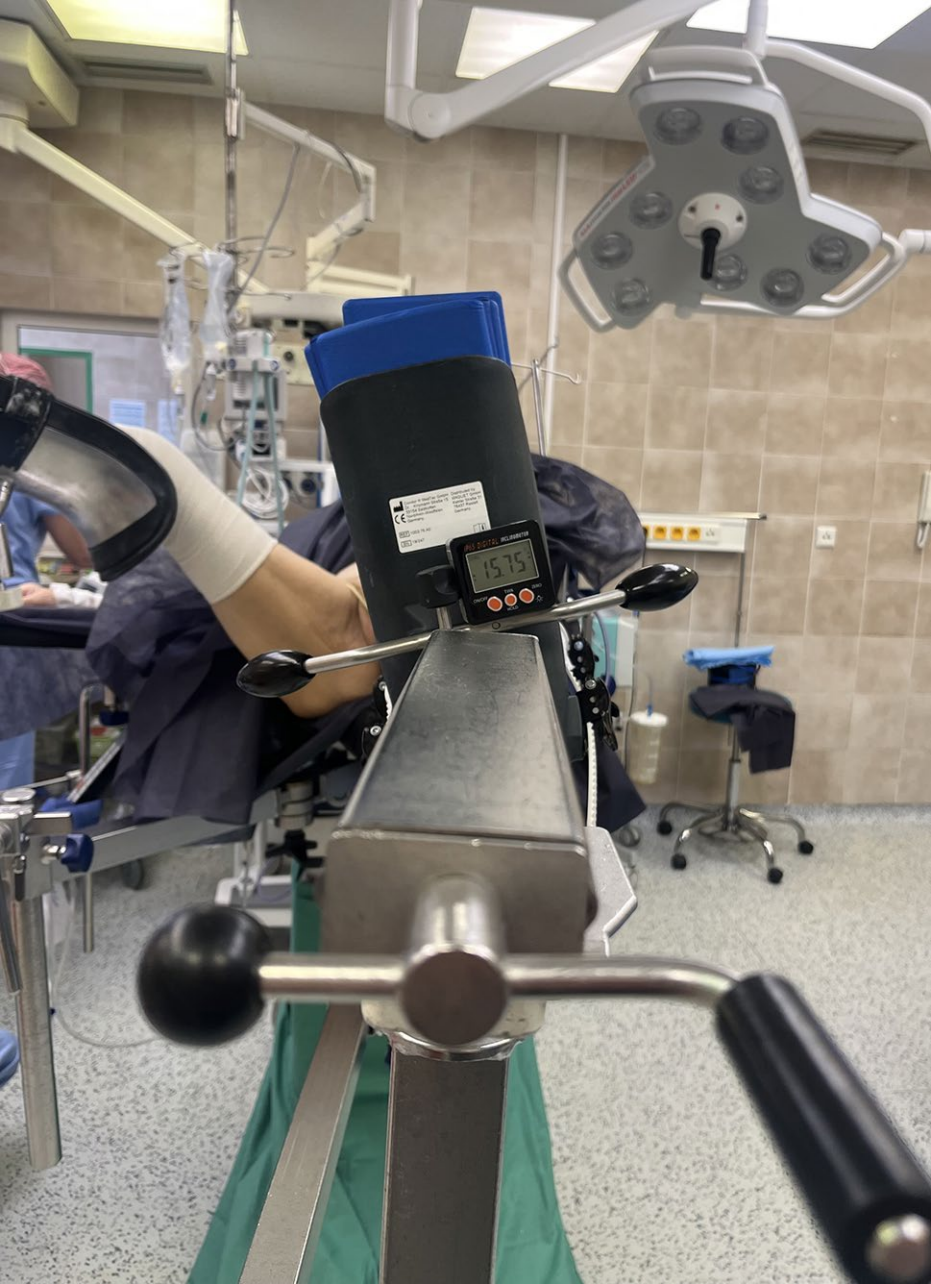
Image of limb rotation measurement on a traction table during closed reduction of a trochanteric fracture

For the surgical procedure, patients were placed in a supine position on the traction table under general or spinal anesthesia. Closed reduction under the fluoroscopy in two perpendicular views was accomplished through traction and rotation of the affected limb. The objective of limb rotation during reduction was to achieve a proper fracture reduction while having the table parallel to the floor. Following the closed reduction on the traction table, we directly measured limb rotation. For exact measurements, to ensure accuracy and mitigate user error, we used a digital inclinometer. This was routinely attached to the traction arm rotating the limb (Fig. 1). From this we obtained an exact value of limb rotation, which the surgeon used for radiologically correct fracture reduction.

For all cases, a single type of a dual lag screw cephalomedullary nail system was used. (PFN Medin, Medin, Nové Město na Moravě, Czech Republic) A short nail was utilized for pertrochanteric fractures (AO 31A1 + 2) and a long nail for intertrochanteric fractures (AO 31A3), with the tip of greater trochanter as an entry point. A dynamic distal locking screw was used in all cases.

Patients meeting the inclusion criteria underwent a low-dose postoperative CT scan (CT CC, Siemens, Erlangen, Germany), capturing both proximal and distal parts of both femurs. The CT scans allowed for the calculation of the angle of femoral version by comparing the orientation of the femoral necks with the tangent passing through the posterior edge of the femoral condyles.

By assessing the difference between both femurs, we determined the malrotation of the affected femur, denoted as the D° value. An increase in the femoral torsion angle indicated internal rotation of the limb (yielding a positive D° value), whereas a decrease signified external rotation of the limb (resulting in a negative D° value).

Intraoperative limb rotation value was compared with the postoperative CT value. We then statistically processed and evaluated the acquired results. We analyzed whether there could be a direct impact of adjusted rotation on final limb malrotation, or if the resulting deformity might be a combination of many often uncontrollable factors, such as fracture type, BMI, individual variable anatomical structure etc. However, if this direct effect proves to be statistically significant, it would allow us to at least establish at least safe thresholds for limb rotation, which would mitigate the error rate to a lesser extent than reported in several previous publications.

### Statistical analysis

Descriptive statistics generated using PYTHON 3.12.3 (by Guido van Rossum, The Netherlands) were utilized for data analysis. For categorical variables, Pearson chi-square tests and Fisher’s exact tests were used to evaluate the significance of differences. *P*-values below 0.05 were considered significant.

## Results

In our cohort of 100 consecutive patients, we observed final malrotation exceeding 15° in 33 patients, corresponding to a 33% error rate. The range of malrotation varied from 28° of external rotation to 39° of internal rotation. In contrast to external malrotation, which was observed only in two cases, internal rotation predominated and was statistically significant. (*p* = 0.001). Within the cohort, we identified 29 A1 fractures, 52 A2 fractures, and 19 A3 fractures. For A1 stable trochanteric fractures, malrotation was observed in 8 patients (25.5%), for A2 multi-fragmentary fractures, malrotation was observed in 18 patients (34.6%) and for A3 fractures in 7 patients were found (36.8%) (Table1). We did not find any significant difference of substantial malrotation while comparing these group of patients based on fracture pattern (*p* = 0.81). The main parameter observed in our study was the relationship between intraoperative limb rotation and final femoral malrotation. Patients were subdivided into five groups based on increments of rotational reduction. The first group included fractures up to 14.9°, followed by groups delineated by five-degree increments (15°–19.9°, 20°–24.9°, 25°–29.9°), with the final group comprising rotations exceeding 30°. In the group of patients with intraoperative rotation up to 14.9°, resultant femoral malrotation was observed in 3 out of 29 patients (10.3%). In the second group with rotations from 15° to 20°, malrotation was noted in 4 out of 20 patients (25.0%). A further minor increase was observed in group 3, where malrotation occurred in 4 out of 14 patients (28.6%). A major increase in incidence of substantial malrotation was evident with intraoperative rotation in group 4 (25°–29.9°). Where final postoperative femoral malrotation was observed in 10 out of 17 of the cases (58.8%) and the difference was significant (*p* = 0,006). Similar findings were noted in patients with intraoperative limb rotation exceeding 30°, with malrotation observed in 12 out of 20 patients (60.0%) (Table 2).

**Table 1:** Evaluated data of the entire study

Baseline data	No. of patients	Dg. of malrotation	(%)	*P* value
Fracture type				0.8631
A1	29	8	27.59	
A2	52	18	34.62	
A3	19	7	36.84	
Anesthesia type				0.0154
General	50	24	48.00	
Spinal	150	43	28.67	
BMI				1.0300
up to 24,9	54	18	33.33	
25 and more	46	15	32.61	
Heathy femur anteversion				0.0979
Up to 9,9	27	16	59.26	
10–19,9	42	10	23.81	
More than 20	31	7	22.58	
Surgeon experience				0.9999
Certified	15	5	33.33	
Uncertified	85	28	32.94

**Table 2:** Comparison of intraoperative limb rotation and postoperative malrotation exceeding 15 degrees. Group stratification

Baseline data	No. of patients	Dg. of malrotation	(%)	*P* value
Leg rotation during surgery				0.0317
0°–14,9°	29	3	10.34	
15°–19.9°	20	4	25.00	
20°–24.9°	14	4	28.57	
25°–29.9°	17	10	58.82	
More than 30°	20	12	60.00	
Comparing groups with peroperative leg rotation				
Up to 14.9°	29	3	10.34	0.0021
15° and more	71	30	42.25	
Up to 19.9°	49	7	14.29	0.0076
More than 20°	51	26	50.98	
Up to 24.9°	63	11	17.46	0.0043
More than 25°	37	22	59.46	

Overall, when evaluating groups with perioperative limb malrotation exceeding 15°, the final rate of malrotation was 42.3% (30/71 patients). This stands in stark contrast to the group with perioperative rotation up to 14.9°, where the final malrotation was found only in 10.3%, indicating a statistically significant difference in malrotation incidence (*p* = 0.00208). When comparing patients with malrotation up to and exceeding 20°, we observed a ratio 14.3% (7/49 patients) vs. 51.0% (26/51 patients) and the difference was significant (*p* = 0.0001). For malrotation exceeding 25°, the percentage increased up to 59.5% (22/37 patients) (Table 2).

Regarding other potential influencing factors, we focused on the impact of anesthesia, building upon our previous study [11] and expanded the sample to a total of 200 patients. Out of the 50 patients operated under general anesthesia, we observed significant malrotation exceeding 15° in 24 cases (48%), while among the rest of 150 patients undergoing spinal anesthesia, malrotation was noted in 43 cases (28.7%). The difference was found to be statistically significant (*p* = 0.0154) (Table 1).

We also investigated potential risk factors for final malrotation. At first, we divided patients according to BMI. Fifty-four patients had a BMI of up to 25, and 18 of these patients exhibited malrotation (33.3%). Among patients with a BMI over 25, 46 patients in total, malrotation was present in 15 cases (32.6%). Here, we did not find a statistically significant difference (*p* = 1.03). When dividing patients according to the physiological ante-version of the contralateral femur, we categorized them into three groups: up to 9.9°, 10°–19.9°, and 20 + °. We did not find a statistically significant difference in error rates with a borderline significance and the lowest error rate observed in patients with the highest anteversion (7/31; *p* = 0.0979). We also examined the potential impact of the surgeon’s experience. In terms of surgeon experience, we set the threshold as certification in the field of Orthopedics, i.e., at least 6 years of experience. We did not find any effect of the surgeon’s experience on rotational error (*p* = 0.9999) (Table1).

## Discussion

Femoral rotational deformities have been extensively investigated in cases of femoral shaft fractures. Previous studies have indicated that malrotation up to 15° is relatively well tolerated due to a femoral neck anteversion compensation [13, 17, 18]. However, larger malrotations can result in persistent hip discomfort, restricted mobility, and subsequent knee instability [17, 22]. A few authors also highlighted a significant risk of degenerative arthritis progression in the knee and hip joints due to femoral malrotation [23, 24]. This condition alters force distribution across adjacent joints, leading to non-physiological loads and uneven stress on the hips and knees.

While the pathological threshold for femoral malrotation is typically considered to be 15°, external rotational errors often remain tolerable, particularly among elderly patients. Nevertheless, there is a growing emphasis on achieving optimal functional outcomes, especially among elderly with high demands from daily-life activities.

Recently, there have been a growing number of articles addressing femoral malrotation following trochanteric fracture fixation with hip nails. Across these studies, the incidence of substantial femoral malrotation after fixation ranges from 20 to 40%, with patient cohorts ranging from 40 to 120 individuals [8–12]. The first reference of functional impairment was published by Kim et al., who assessed VAS and Koval scores among patients with malrotation [9]. However, their study did not identify any statistical differences in functional outcomes when comparing patients with or without malrotation. A very recent study by Pande et al. focused on the impact of malrotation and observed significant differences in Harris Hip Scores (HHS), Oxford Hip Scores (OHS), Oxford Knee Scores (OKS), and Visual Analogue Scores (VAS) upon detailed monitoring of patients for up to 24 months. Among 20 patients with significant malrotation, they reported pathologies including gait abnormalities, significant deterioration of knee function, significantly reduced ability to squat and sit cross-legged, and an increased demand for assistive devices [25].

Few studies have focused on a potential direct effect on the occurrence of malrotation during the osteosynthesis of trochanteric fractures. Yurteri et al. searched for the impact of the patient’s perioperative position [19]. They compared the supine position on a traction table with the lateral decubitus position. Despite noting the advantages of the lateral decubitus position in terms of the absence of a traction table and lower radiation exposure for the surgeon, no statistically significant difference in the resulting femoral malrotation was observed.

Our study dealing with other factors was published in 2023 [11]. We examined the impact of the surgeon’s experience, acute vs. delayed surgery, fracture type, and the type of anesthesia. We found in a cohort of 100 patients a lower rate of malrotation in patients operated under spinal anesthesia compared to those operated under general anesthesia and this result approached statistical significance with a borderline value. However, while adding another 100 examined patients in our recent study, the difference was deemed statistically significant (*p* = 0.0154). We attribute this phenomenon to the higher traction mainly by the hip rotators and inadequate patient relaxation under general anesthesia. We hypothesize decreased relaxation may result in poorer maneuvering ability thereby complicating the reduction process.

Intraoperative measurement of femoral anteversion is highly challenging in trochanteric fractures. Auxiliary methods commonly used in femoral shaft fractures, such as the shape of the lesser trochanter, the width of adjacent cortices, orientation of the distal and proximal femur etc., cannot be relied upon here. The study by Kinami et al. yielded very precise results measuring the femoral neck anteversion using a digital goniometer on an iPhone application [20]. Perioperative measurements obtained with this technique were compared with postoperative CT scan results. The findings showed minimal differences between the two methods, indicating high accuracy and reliability of the proposed approach. The results revealed a median difference of 3.0° between axial-projected lag-screw anteversion (AxP-LS-AV) and measurements obtained from 3D-CT (three-dimensional computed tomography). Moreover, 80% of patients exhibited differences of 5° or less between these two measurement methods. However, the authors themselves mentioned drawbacks of this method during measurements, including prolonged surgical time, increased radiation exposure, and the necessity of obtaining measuring equipment. The utilization of the method would also require precise knowledge of the anteversion of the healthy contralateral femur using preoperative CT imaging. In the continuation and further investigation of this technique, as stated in their subsequent publication, they abandoned it because of the lack of accuracy in measuring the angle between the lag screw guide pin and the neck axes [21]. However, they smoothly transitioned their research by using direct intraoperative measurement of femoral anteversion with standardized hip and knee imaging. The authors applied similar imaging techniques with high accuracy, as used by Ivanov et al. in their cadaver study, with very promising results [26]. According to the initial results, this method appears to be sufficiently accurate when compared to postoperative CT imaging. As the authors mentioned, it as well certainly leads to an extension of the operation time, changes in the standard patient positioning, as well as inter-individual variation in assessment between different surgeons. Additionally, it requires significant practice in very precise assessment of fluoroscopy during the procedure. Therefore, despite the high accuracy of the method, it is questionable whether it can be widely adopted into routine practice or should be reserved for complicated cases.

**Fig. 2:**
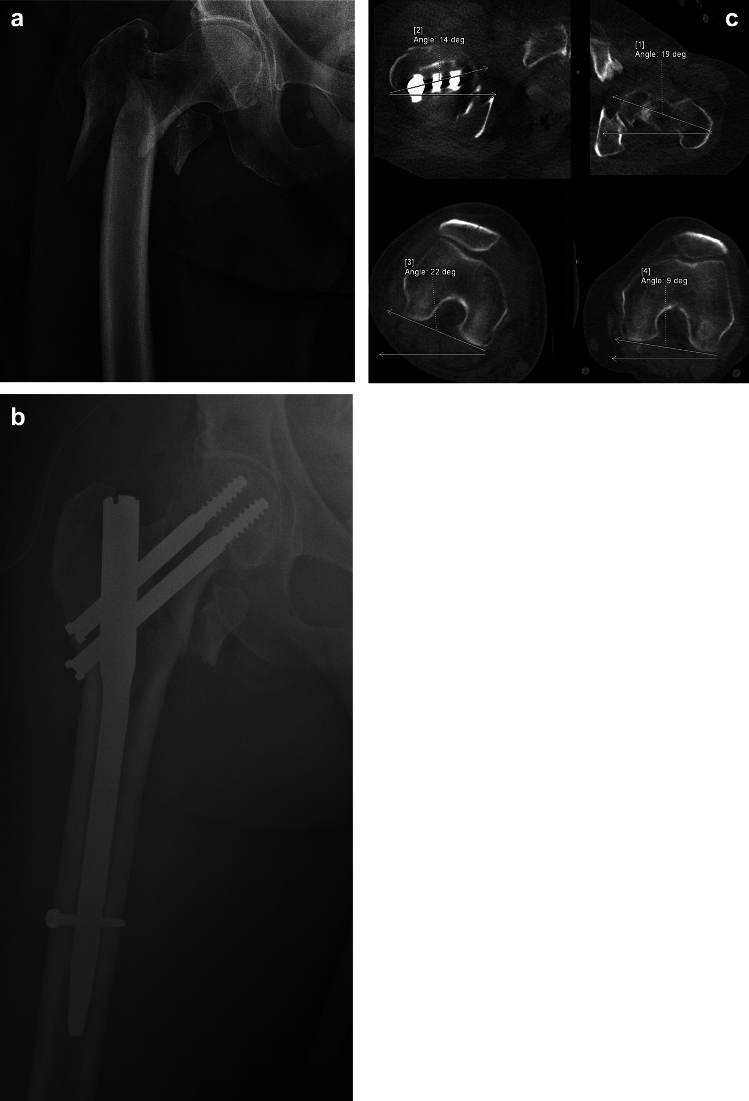
**a** 78y old female, trochanteric fracture AO 31-A3.3, **b** Performed osteosynthesis with a short nail in an apparently satisfactory position, **c** Postoperative malrotation of the operated limb—26° internal rotation compared with the healthy limb

In our study, we investigated whether there is a direct influence of limb rotation on the traction table on the resulting malrotation of the operated femur. Naturally, we are aware of a whole range of other factors that may have a direct or indirect impact on the femoral malrotation, as well as limitations in measuring limb rotation on the traction table including knee laxity and the boot fixation properties. These factors include, but are not limited to, the type of fracture, BMI, patient musculature, laxity of surrounding joints, history of previous lower limb surgery, and the type of anesthesia. There is a wide array of variables that almost preclude us from determining the precise or even approximate femoral neck anteversion in a straightforward manner; surgeons usually attempt to maintain the limb in a neutral position based on the orientation of the patella. However, this technique is still unreliable due to inter-individual anatomical variations of knee and patella itself. Despite many studies, we observe that the rate of malrotation after surgery remains very high [8–12]. Based on our experience, surgeons often tend to exaggerate the rotation in order to achieve proper fracture alignment, yet postoperative CT scans can reveal excessive malrotation. (Fig. 2a–c).

We have found that clear guidelines for the reduction of trochanteric fractures that have not been published yet. Already within the range of 15–20° of rotation, the incidence of significant malrotation rises from 10 to 25%. Within the range of 20–25°, it further increases to 31%. The threshold of 25° of internal rotation had the most statistically significant observation where the incidence of malrotation reached almost 60% and the rate of severe malrotation was doubled.

We are aware of the limitations of the study, stemming from the smaller number of patients, and that we must work with a certain degree of statistical error. It will be necessary to supplement the study with a follow-up study involving a larger number of patients to in order to refine our conclusions.

In our cohort, no patient required closed reduction by external rotation. Therefore, these results suggest a relatively straightforward recommendation: internal rotational up to 20° is considered to be within the safe zone, but when the reduction exceeds 20°, the risk of substantial malrotation is doubled and therefore unacceptable. The measurement of limb rotation on the traction table, which only takes few seconds using a standard goniometer, is cheap and effective in preventing higher malrotational errors and avoiding serious consequences. Moreover, operating under spinal anesthesia appears to be favorable in achieving proper reduction by easy rotation.

## Conclusions

Improper reduction of a trochanteric fracture is a common problem. Excessive rotation on the traction table in an attempt to achieve better fracture alignment significantly increases the risk of femoral malrotation. Based on our study, the limb should not be rotated more than 20° on the traction table to minimize the risk of postoperative femoral malrotation, as we consider that every second patient will then experienceclinically significant malrotation exceeding 15°. We recommend standard use of goniometer for the measurement the reduction in rotation. We also highlight the advantage of spinal anesthesia over general anesthesia due to a significant reduction in postoperative femoral malrotation.

## Data Availability

The data that support the findings of this study are not openly available due to reasons of sensitivity and are available from the corresponding author upon reasonable request. And can be send wia electronic storage system.
